# *Periploca forrestii* Saponin Ameliorates Murine CFA-Induced Arthritis by Suppressing Cytokine Production

**DOI:** 10.1155/2016/7941684

**Published:** 2016-12-12

**Authors:** Yingqin Liu, Minghui Li, Qiuhong He, Xinping Yang, Fang Ruan, Guangchen Sun

**Affiliations:** ^1^Biotechnology College, Guilin Medical University, No. 109 North 2nd Huan Cheng Road, Guilin, Guangxi 541004, China; ^2^Pharmaceutical College, Guilin Medical University, No. 109 North 2nd Huan Cheng Road, Guilin, Guangxi 541004, China

## Abstract

*Periploca forrestii* Schltr. has been used as a Chinese folk medicine due to its versatile pharmacological effects such as promoting wounds and rheumatoid arthritis. However, the antiarthritic activity of* Periploca forrestii* saponin (PFS) and its active compound Periplocin has still not been demonstrated. Here, we evaluated the antiarthritic effects of PFS in adjuvant-induced arthritis (AIA) rats by intragastric administration at a dose of 50 mg/kg. The anti-inflammatory activities of Periplocin were also examined in LPS-induced AIA splenocytes and synoviocytes. PFS significantly ameliorated joint swelling; inhibited bone erosion in joints; lowered levels of IL-6 and TGF-*β*1 in AIA rat splenocyte; and reduced joint protein expression levels of phospho-STAT3 and IKK*α*. Using LPS-induced AIA splenocytes, we demonstrate that Periplocin suppressed the key proinflammatory cytokines levels of IL-6, IFN-*γ*, TGF-*β*1, and IL-13 and IL-22 and transcription factor levels of T-bet, GATA3, and C-Jun genes. Periplocin also suppressed LPS-induced cytokine secretion from synoviocytes. Our study highlights the antiarthritic activity of PFS and its derived Periplocin and the underlying mechanisms. These results provide a strong rationale for further testing and validation of the use of* Periploca forrestii* Schltr. as an alternative modality for the treatment of RA.

## 1. Introduction

Rheumatoid arthritis (RA), which afflicts about 1% of the world's population, is a systemic, chronic, autoimmune inflammatory disease that preferentially attacks the synovial lining of the joints, destroys local articular structures, and further affects related tissues and organ systems [1, 2]. Untreated RA leads to deformities and disability [3]. Therefore, therapy to joint inflammation and cartilage destruction is a logical strategy for preventing the progression of RA [4, 5].

A major contributor to joint inflammation in RA and antigen-induced arthritis (AIA) is proinflammatory cytokines. IFN-*γ*, IL-6, and IL-22 are found to be elevated during the development of arthritis, and inhibition of TNF and IL-6 represents successful treatments of RA [6, 7]. Conversely, anti-inflammatory cytokines (e.g., IL-10, TGF-*β*, and IL-13) may dampen arthritis [3].

Despite this clear link between inflammation and increased bone turnover in RA and the existence of several therapeutical options, their efficacy on inflammation and bone treatment seem to be uncoupled, with some drugs suppressing inflammation but failing to protect bone [8, 9] and others halting bone destruction but with no effect on controlling inflammation [10].

The dry root or whole vine of* Periploca forrestii* Schltr. of Asclepiadaceae is effective in clinical prescription for promoting blood flow and eliminating wind effect cardiac antitumor and anti-inflammation, widely used in the treatment of rheumatoid diseases [11, 12]. Periploca forrestii mainly contain cardiac glycosides, flavonoids, C21 steroid saponins and other triterpenoid ingredients. Saponins are the characteristic components and also the main active ingredients of* Periploca forrestii*. Periplocin is one of cardiac glycosides extracted from* Periploca forrestii*, several studies have addressed the various heart conditions [11–14]. Recent studies also suggest that Periplocin extracted from cortex periplocae can inhibit cell growth in colon cancer cells, lung cancer cells, and hepatocellular carcinoma cells [15–17].

Our aim in the herein study was to test the effect of PFS treatment in the gene expression of inflammatory cytokines and on the overall synovial tissue joint structure in a rat AIA model, as a further argument to its possible efficacy in RA treatment. In this work we also report that Periplocin significantly decreases cytokines in AIA splenocytes and normal synoviocytes.

## 2. Materials and Methods

### 2.1. Reagents and Animals

Periplocin was purchased from State General Administration of the People's Republic of China for Quality Supervision and Inspection and Quarantine. p-Stat3, IKK*α*, and p-I-*κ*B*α* were purchased from Santa Clauz. Female Sprague Dawley rats (6–8 weeks old) with a mean weight of 150–180 g were obtained from Laboratory Animal Center of Guilin Medical University, China. Rats were housed in an appropriate environment with an air-filtering system. All experimental procedures were approved by the Research Ethics Committee of Guilin Medical University, China.

### 2.2. Collection of* Periploca forrestii* and Saponin Extract Process

*Periploca forrestii* were collected from Guangxi province, China, and identified by Dr. Sun from Guilin Medical University. The powder obtained by mincing dried roots was extracted for 2 h with 70% ethanol. The extract was reextracted twice following the same procedure and then filtered. The filtrate was partitioned with petroleum ether (1 : 1) and then butanol. The butanol solvent was evaporated in a vacuum evaporator to yield the butanol fraction.

The final extract was concentrated and dried to obtain PFS. To study the antiarthritic activity of PFS, the butanol extract was dissolved in water and arthritic Sprague Dawley rats were fed at the dose of 50 mg/kg body weight (normalized from the amount of human dose) by using the regimen described below.

### 2.3. Induction of Arthritis

Female Sprague Dawley rats were randomly divided into the following three groups: normal group = normal rats; AIA control group = CFA-induced arthritis rats; FPS group = PFS 50 mg/kg treated AIA rats. Chronic arthritis was induced by five-point injection of total of 0.5 ml of Freund's complete adjuvant (CFA, Sigma Aldrich) containing 1 mg of heat killed* Mycobacterium tuberculosis* in 1 mL of mineral oil into the back skin of the Sprague Dawley rat intradermally. Two weeks later, arthritis was induced by subplantar administration of 0.1 ml of CFA into both hind paws of all the rats, respectively. Before 7 days of adjuvant injection in paws, immunized groups were orally given with 50 mg/kg/day until the day before tissue harvesting, 28th day. Control group were given water. The thickness of the paw was measured before induction using a dial thickness gauge (Peacock Japan) and after treatment.

### 2.4. Histology, and Immunohistochemistry

The hind paws were harvested from rats on day 28 after CFA immunization. Each paw was fixed in 4% paraformaldehyde overnight and then demineralized with 10% ethylenediaminetetraacetic acid (EDTA) for one month before embedding in paraffin. Paraffin-embedded tissue was serially sectioned at 5 *µ*m distances serially using a microtome and mounted on microscope slides. Then the sections were stained either with toluidine blue and fast green or with safranin-O (safranin-O stain is taken up by the intact cartilage), Tartrate Resistant Acid Phosphatase (TRAP), or Matrix Metalloproteinase 11 (MMP-11). Histopathological changes in the joints like synovial hyperplasia, pannus formation, and cartilage erosion and bone erosion were observed under a microscope using the Spot Imaging Software, and Representative photos were taken with an Olympus microscope at 100x magnification.

### 2.5. Western Blotting

Frozen synovial tissue (whole joints including synovium, adjacent tissues, and bones) was weighed and broken into pieces on dry ice. Paw lysates were homogenized in Radio Immunoprecipitation Assay (RIPA) lysis buffer (50 mM Tris-HCl, 150 mM NaCl, 1% Triton X-100, and 1% deoxycholate) supplemented with 15 mM sodium fluoride, 1 mM sodium vanadate, 2 mM sodium pyrophosphate, 1 mM sodium glycerophosphate, 2 mM imidazole, 100 mg/ml phenylmethylsulfonyl fluoride and proteinase inhibitor cocktail (Sigma Aldrich), and sonicated in the lysis buffer, cleared by centrifugation at 12,000 g for 10 minutes at 4°C. The protein samples were isolated from the inflamed paw tissue and quantified using Bradford assay and kept at −80°C until use. The protein samples (25 *μ*g/well) were electrophoresed by using 10% sodium dodecyl sulfate- (SDS-) polyacrylamide gel electrophoresis (PAGE) and transferred to polyvinylidene fluoride membrane. Immunoblot analysis was carried out using rabbit anti-rat polyclonal antibody (1 : 1000) as primary antibody and Horseradish Peroxidase (HRP) conjugated goat anti-rabbit IgG (1 : 5000) as secondary antibodies, respectively. The proteins were detected using enhanced chemiluminescence (ECL). The band intensity was measured densitometrically using Image J software. *β*-Actin was used to normalize the target and the relative expressions were stated as fold ratio.

### 2.6. Preparation of Splenocytes and Their Restimulation with LPS for Cytokine Testing

Sprague Dawley rats were injected 100 *μ*l CFA (1 *μ*g/ml) under the right plantar inflammation intradermally; two weeks later, splenocytes were isolated. After erythrocytes were removed from spleen cell suspensions using red cell removal buffer 0.16 M Tris-NH_4_Cl solution, splenocytes in the suspensions were adjusted to a cell density of 2 × 10^6^ cells/ml. These spleen cells were placed in a 96-well plate at 37°C in Dulbecco minimum essential medium (DMEM) supplemented with 5% fetal bovine serum (FBS), 2 mM L-glutamine, 100 units/ml penicillin G sodium, and 100 *μ*g/ml streptomycin sulfate. Splenocytes (100 *μ*l) were added to each of the wells of a 96-well plate in the presence or absence of 100 *μ*l of final concentrations of 2 *μ*g/ml periplogenin and lipopolysaccharide (LPS) (1 *μ*g/ml). The cells were incubated at 37°C in a humidified atmosphere of 5% CO_2_ for 3, 6, 12, and 24 h.

### 2.7. Synoviocyte Culture

Synovial tissues were obtained from the knee joints of normal Sprague Dawley rats. The synovial tissues were minced and stirred with type IV collagenase (Sigma Aldrich Co., USA) in serum-free DMEM/F12 medium at 37°C for 2 h in an incubator shaker. The synovial tissue lysate was then filtered through a 40 *μ*m nylon mesh, washed extensively, and seeded in 12-well microplates. The cells were cultured in DMEM/F12 supplemented with 10% FBS and benzylpenicillin potassium (100 units/ml) at 37°C/5% CO_2_.

### 2.8. Quantitative Expression for Target Genes

The total RNA of spleen or cell was isolated using TRIzol reagent according to the manufacture's instruction (TRIzol, Invitrogen). The purity and yield of RNA were assessed by measuring the absorbance of RNA solution at 260 nm and 280 nm. The RNA product sizes were estimated relative to 100 bp DNA ladder. cDNA was synthesized from 1 *μ*g of RNA with 2x Tak PCR Master Mix RT mix (Aidlab Biotechnologies) on the final volume of 20 *μ*L according to manufacturer's guidelines. The specific forward and reverse primers were used for target genes expression (Table 1). Beta-actin was used to normalize differences in RNA isolation, RNA degradation, and the efficiencies of the reverse transcription. The relative expression of target genes was stated as fold ratio using Smart View Method.

### 2.9. Statistical Analysis

All experimental results are expressed as mean (SD) of several independent experiments. Multiple comparisons of data were carried out by analysis of variance (ANOVA) with Dunnett's test. *p* values of less than 5% were regarded as significant.

## 3. Results

**Figure 1:**
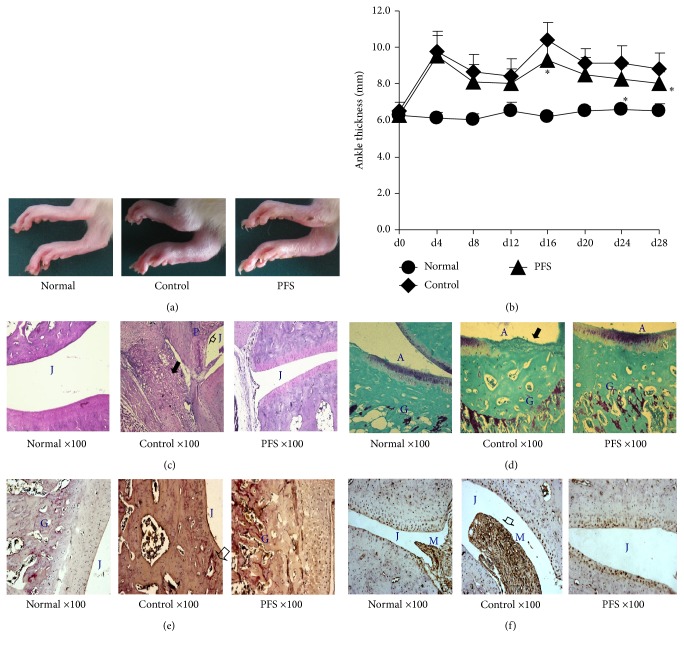
PFS prophylactically suppress inflammation in CFA-induced arthritis in rats. AIA was induced as detailed in Methods. Rats were randomly divided into 3 treatment groups: normal, control, and PFS. PFS were administered orally until the day before tissue harvesting 28th day. (a) Representative photographs of days 21–27 paws from the 3 treatment groups. (b) Change in ankle thickness. The changes in paw thickness were measured every four days. The results are expressed as the mean ± standard error (*n* = 7–9). Statistical values conducted on days 0–28 for changes in paw thickness compared with control were as follows: ^*∗*^ *p* < 0.05 compared with the control group, Dunnett's test. ((c)–(f)) Representative photographs of knee joint tissues stained with safranin-O-fast green, toluidine blue-fast green, TRAP, or MMP-11 (magnification, 100x). Note that the intense synovial inflammatory infiltration ((c) black arrowhead), pannus formation ((c) arrowheads), cartilage destruction, extensive proteoglycan depletion ((d) black arrowhead), and MMP-11 infiltration ((f) arrowhead) were significantly reduced in the joints of PFS-treated rats compared with the respective control rats. A: articular cartilage; G: growth plate; J: joint space; M: synovial membrane; P: pannus formation.

In the present study, we investigated the in vivo efficacy of PFS in AIA rats. Each group of Sprague Dawley rats was fed daily with PFS starting before CFA immunization and then continued for 35 days. The control rats received water orally. Rats immunized with 100 *μ*g CFA began to develop arthritis in the first week. Then the initial manifestation of arthritis was erythema and swelling of ankle joints, followed by the inflammation of the metatarsal and interphalangeal joints. Disease progression can be evaluated by measuring paw swelling thickness, which is an indicator of the degree of inflammation. In order to evaluate the antiarthritic efficacy of PFS, the paw thickness changes were quantified using Peacock thickness meter. PFS significantly ameliorated paw swelling. At the end of the experiment, more significant reductions of paw thickness were observed in groups treated with PFS (50 mg/kg) (Figures 1(a) and 1(b)).

Effects of PFS on joint histopathological changes of experimental animals were examined by safranin-O, toluidine blue, TRAP, and MMP-11 staining. Representative microphotographs were presented for normal, control, and PFS-treated group. Normal animals had shown no detectable abnormalities; for the control arthritic rats; the knee joints had moderate evidence of articular cartilage damage with pannus formation. The formation of pannus is a result of overgrowth of the synoviocytes and the observed accumulation of inflammatory cells that led to deformed cartilage and bone. The synovial membrane and capsule were both markedly thickened as a result of pannus formation and inflammatory cell infiltration (Figure 1(c)). In control group, the chronic inflammation destroyed the joint lining, including extensive proteoglycan depletion cartilage and other nearby supporting structures, such as bone (Figure 1(d)). At some locations in AIA joints osteoclasts resorbed the subchondral bone plate up to the noncalcified cartilage (Figure 1(e)). The infiltration of MMP-11 was found in hyperplastic synovial linings with sloughing of synoviocytes into the joint space (Figure 1(f)). In addition, these changes were ameliorated in PFS-treated animals. These results show that PFS have antiarthritic activity.

**Figure 2:**
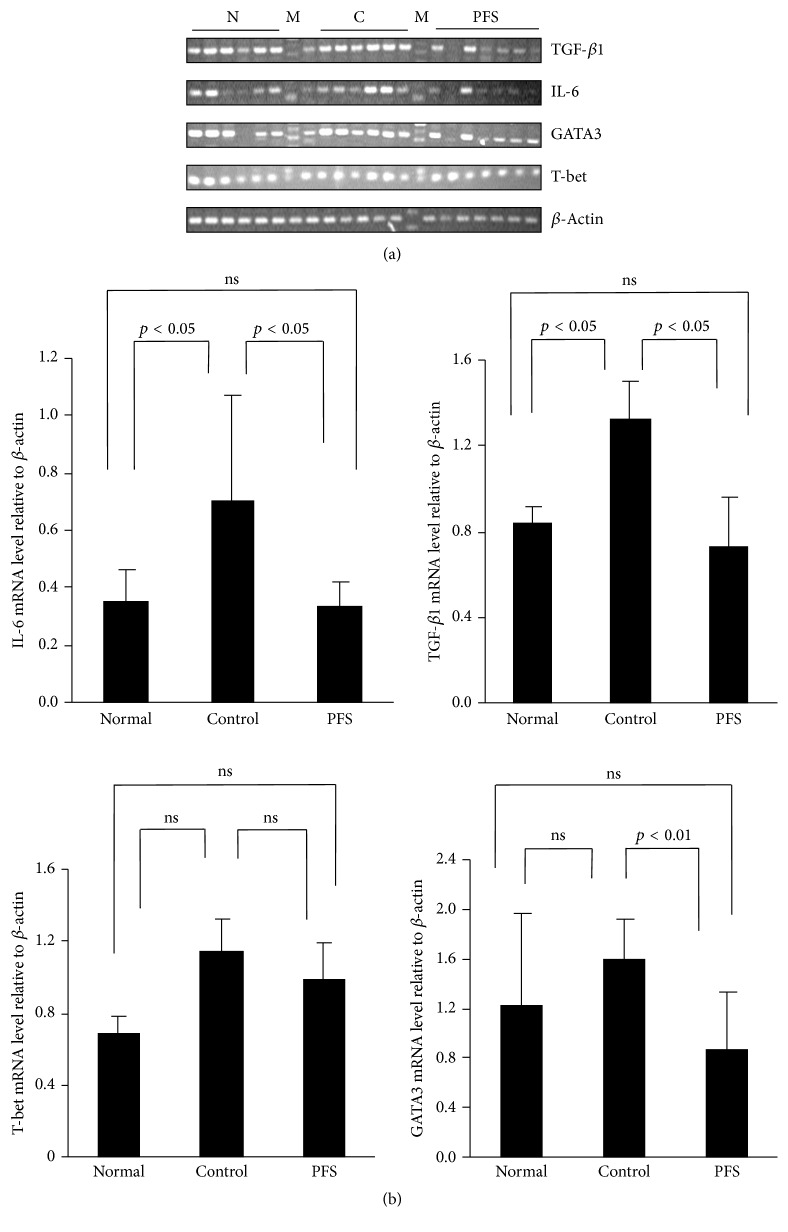
PFS suppressed the expression of inflammatory cytokines. AIA was induced as detailed in Methods. Arthritic rats were treated with 50 mg/kg PFS for 35 days. Day 28: splenocytes were harvested for measuring cytokine expression by quantitative RT-PCR. (a) RT-PCR analysis of inflammatory cytokine genes and related transcription factors and (b) relative amounts of each cytokine gene level were determined by densitometric analysis. Dunnett's test. N: normal; C: control; M: marker. ns: no significant difference.

We performed in vivo assay of CFA-induced cytokine production by rat splenocytes to clarify that whether PFS treatment regulates cytokine production. Cytokine levels were measured in the spleen tissues to obtain insight into the mechanisms of beneficial PFS-mediated effects. In Figure 2, the reverse-transcription polymerase chain reaction (RT-PCR) results indicated the presence of relatively small amounts of IL-6 and TGF-*β*1 mRNA in the spleens of healthy rats. However, the concentrations of these transcripts exhibited a substantial increase in the joints of the rats with AIA. There was a significant decrease (*p* < 0.05) in the expression of TGF-*β*1 and IL-6 mRNA in PFS-treated rats compared with their respective controls. However, the decline in the T-bet mRNA level in PFS-treated rats was not significant. These results suggested that PFS regulated systematic immunological response by simultaneously reducing IL-6 and TGF-*β*1 production and transcription factors at spleen in rats with RA.

**Figure 3:**
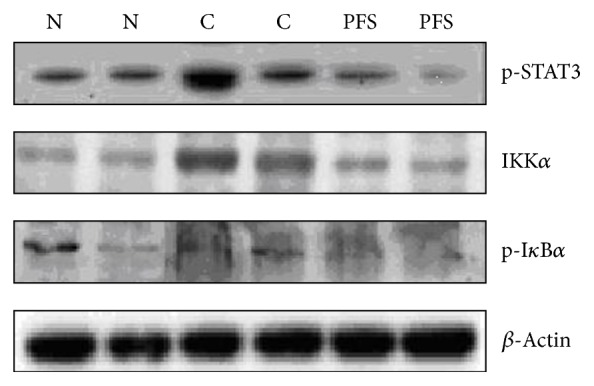
Effect of PFS treatment on protein expression levels of p-STAT3, IKK*α*, and p-I*κ*B*α* protein expression levels in the AIA rat model. AIA was induced as described in Methods. Arthritic rats were treated with 50 mg/kg PFS for 35 days. In day 28 off-target organs paws were processed and protein lysates were probed for expression of p-STAT3 and related NF-*κ*B family members (IKK*α* and p-I*κ*B*α*) by Western blotting. N: normal; C: control.

As STAT3 has crucial roles in inflammation, the regulatory effects of PFS on the inflammatory response in the AIA rats were examined. p-STAT3 protein expression was elevated in the AIA rats compared with that of the normal group (Figure 3). Notably, PFS administration at 50 mg/kg significantly reduced p-STAT3 and IKK*α* protein expression in the RA rat model.

**Figure 4:**
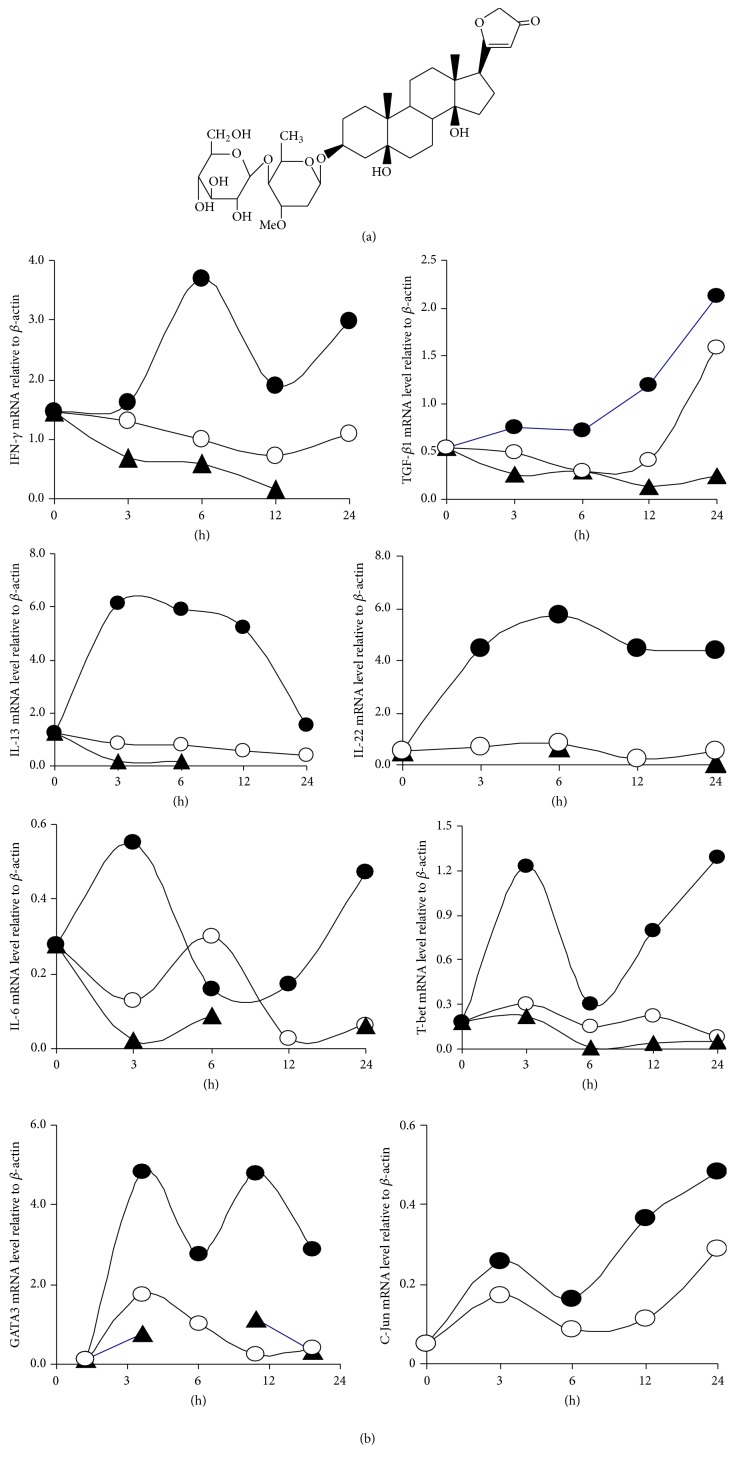
Effect of Periplocin on proinflammatory cytokine expression in splenocytes from Freund's complete adjuvant Sprague Dawley rats. Splenocytes were prepared as described under Methods. Splenocytes were cultured in the presence or absence of Periplocin or LPS for indicated time. The total mRNA was prepared for RT-PCR analysis. (a) Chemical structure of Periplocin, (b) RT-PCR analysis of inflammatory cytokine genes and related transcription factors, and relative amounts of each cytokine gene level were determined by densitometric analysis. L: LPS; P: Periplocin; ●: LPS only; ○: LPS + P; ▲: P only.

LPS is a major component of the cell wall of Gram-negative bacteria and is a well-known potent inducer of inflammation and inflammatory bone loss [18]. We prepared splenocytes from Freund's complete adjuvant Sprague Dawley rats. LPS-induced production of proinflammatory factors IL-6, Th1 (IFN-*γ*), Th2 (TGF-*β*1 and IL-13), and Th17 (IL-22) and transcription factors T-bet, GATA3, and C-Jun mRNA were increased in splenocytes harvested. However, the mRNA levels were reduced in a time-dependent manner in Periplocin-treated splenocytes (Figure 4).

**Figure 5:**
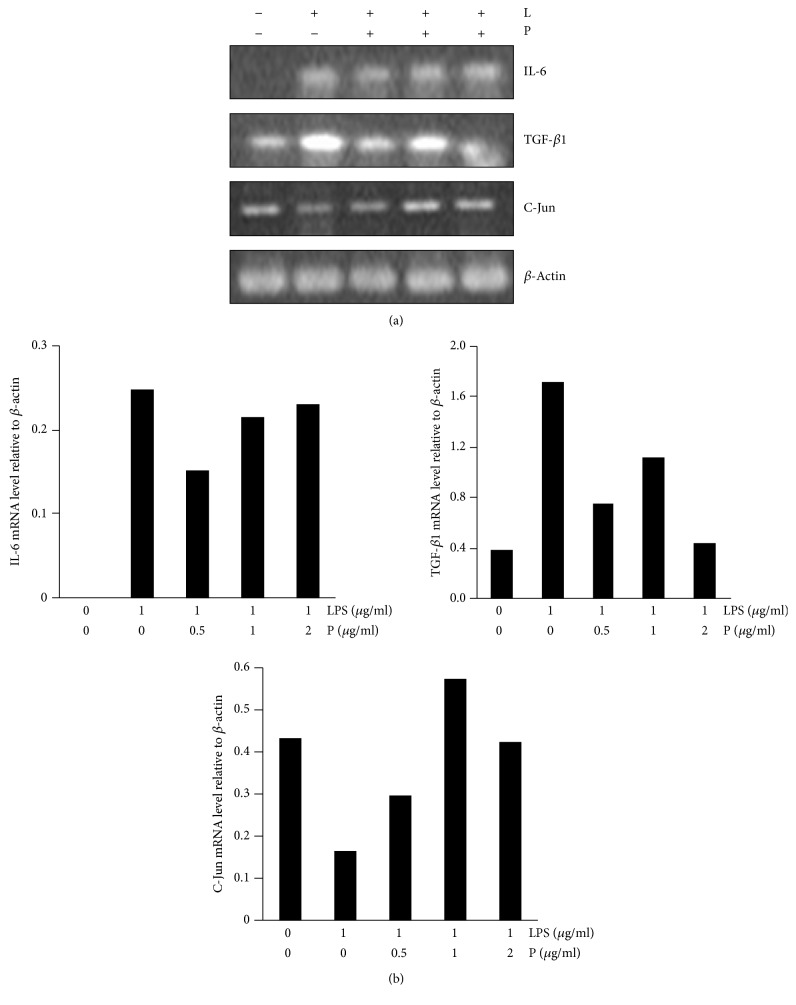
Effect of Periplocin on proinflammatory cytokine expression in synoviocytes from normal Sprague Dawley rats. Synoviocytes were prepared as detailed in Methods. Synoviocytes were incubated with the indicated concentrations of Periplocin for 24 h in the presence or absence of LPS (1 *μ*g/mL) stimulation. After 24 h, the mRNA was measured by RT-PCR and *β*-actin was used as the internal controls, respectively. Representative gels stained for RT-PCR products of IL-6 and TGF-*β* mRNA expression of synoviocytes in AA model rats treated with Periplocin. (a) RT-PCR analysis of inflammatory cytokine genes and related transcription factors and (b) relative amounts of each cytokine gene level were determined by densitometric analysis. Steady-state expression of *β*-actin was used to control equal loading of the PCR product onto gels. The histogram shows the mRNA levels. B: blank; L: LPS; P: Periplocin.

LPS can stimulate FLS to secrete MMPs, and this induction is regulated at the transcriptional and translational levels [19]. Effect of different concentrations of mRNA expression of IL-6, TGF-*β*1, and C-Jun in LPS-induced synoviocytes was examined. In the blank group, there was weak mRNA expression of IL-6 and TGF-*β*1, while the expression of cytokines was significantly increased in the LPS group. As the dose of Periplocin gradually increased from 0.5 *μ*g/ml to 2 *μ*g/ml, mRNA expression of IL-6 and TGF-*β*1 was reduced in a concentration-dependent manner. While C-Jun level was reduced in LPS-induced synoviocytes, however, increased C-Jun level was found in a concentration-dependent manner (Figure 5).

## 4. Discussion

AIA is characterized by a rapid onset and progression to articular inflammation. Usually the disease is severe and leads to permanent joint malformations, including ankylosis. Symmetric joint involvement, lymphocyte infiltration, cartilage degradation, synovial hyperplasia, and T cell dependence are shared features with human RA [20, 21]. Subdermal injection of FCA at multiple sites around the tibiotarsal joint of female Sprague Dawley rats caused a localised inflammatory reaction to develop in 24 h. A striking feature was the presence of synovial thickening with foci of cartilage erosion and a prominent bone destruction around the ankle joint.

Many potent antiarthritic drugs are available for the management of arthritis. However, their prolonged use is associated with severe adverse effects and they are ineffective in a proportion of patients. In addition, they are expensive. However, their prolonged use is associated with severe adverse effects. Accordingly, an increasing number of patients with RA and other diseases in developed countries are using natural products and other complementary and alternative medicine (CAM) approaches for their healthcare needs [22–26].

A variety of herbs belonging to traditional Chinese medicine have been used in China for centuries for the treatment of rheumatic diseases, including RA. Many studies suggest that traditional herbal resources benefit the management of inflammatory arthritis and may therefore benefit RA. Many Chinese herbs recorded effective in the therapy of RA have been analyzed and the related mechanisms have been further detected [27].

*Periploca forrestii* shows several medicinal prophylactic effects in traditional Chinese folk medicine; because several plant saponins were recently found having therapeutic effects in different models of autoimmune diseases, such as arthritis [28, 29], we suggest that PFS may have clinically beneficial effects on autoimmune diseases by their induction of immunosuppression during chronic administration. Therefore, the present study was conducted to evaluate the anti-inflammatory and antiarthritic activities of PFS based on an experimental model of autoimmune arthritis (AIA model).

In this study, PFS alleviated the clinical outcomes, synovial hyperplasia inflammatory cells infiltration, and cartilage destruction in CFA rats. It revealed that PFS inhibited inflammatory cytokines and the transcriptional activity by suppressing STAT3 signaling. The splenocytes of CFA-immunized Sprague Dawley rats treated with PFS showed a significantly reduced expression of TGF-*β*1 and IL-6 and further GATA3, but a slight effect on T-bet. The relative lack of effect on T-bet suggested that PFS had a major effect on the activity of the differentiation of naive T cells into Th2 cells, but not Th1 cells. To address whether PFS modulated the NF-*κ*B signaling pathway, we attempted to analyze the expression of NF-*κ*B signaling transduction proteins in the absence or presence of PFS. We showed that CFA induced phosphorylation of STAT3 and that PFS inhibited the effect. Furthermore, it has been shown that IL-6 can induce the phosphorylation of STAT3 in certain cell lines [30]. In this regard, it is likely that the reduced paw p-STAT3 observed by Western blotting in our study might be attributable both to a direct effect of PFS on STAT3 and to an effect via reduced IL-6 expression. These data indicate that PFS might improve AIA by modulating STAT3-regulated genes and thus affects various biological events. The precise mechanisms involved remain to be tested.

As no studies have been conducted to evaluate the efficacy of PFS for the treatment of RA, it is difficult to perform advanced mechanistic and specificity of action studies using a crude plant extract, which possesses multiple components. It is rationale for examining the mechanism of action of Periplocin derived from the plant* Periploca forrestii* Schltr., which then could be extrapolated to that of the natural PFS. Therefore, the present study was conducted to evaluate the anti-inflammatory and antiarthritic activities of PFS active ingredient Periplocin on LPS-induced AIA splenocytes and on LPS-induced synoviocytes. Most of the studies on Periplocin are based on its activity on circulatory system and tumor disease. Our study has unraveled the immunological basis of the antiarthritic property of this naturally occurring plant compound and most of the mechanistic attributes of Periplocin are similar to those of PFS.

Because LPS stimulated many TGF-*β*1 and IL-6 production by splenocytes (Figure 5), we considered whether the TGF-*β*1 and IL-6-inhibiting activity of PFS might correlate with immunoregulation activity. We found that Periplocin derived from PFS had relatively high immunoregulation activity. Indeed, it has been demonstrated that Periplocin reduces the levels of RA factors IL-6, Th2 cytokines (TGF-*β*1 and IL-13), Th1 (IFN-*γ* and IL-33), and Th17 (IL-22) and inhibits the expression of GATA3, T-bet, and C-Jun in LPS-induced splenocytes. Similar results were also found in LPS-induced synoviocytes by suppressing the IL-6 and TGF-*β*1 expression; Periplocin has thus a broad spectrum of targets, modulating not only immune cytokines but also relative transcription factors.

Cytokines are directly implicated in many of the immune processes that are associated with the pathogenesis of rheumatoid arthritis. There are several studies comparing circulating levels of cytokine; they often show discrepancy in their results. Similar to our AIA, serum IL-6 levels are substantially increased in RA with significant circadian variations corresponding to the circadian rhythm of symptoms in RA [31]. Studies addressing the role of TGF-*β* and IL-10 in experimental arthritis have shown variable results. Significantly elevated TGF-*β*1 levels have been reported in serum of RA patients [32]. Anti-TGF-*β* and anti-TGF-*β*RI antibodies injection inhibits chronic synovial inflammation in rats with streptococcal cell wall-induced arthritis [33] and in antigen- and collagen-induced arthritis mice [34]. In this study, the splenocytes of CFA-immunized Sprague Dawley rats lead to increased TGF-*β*1 levels in vivo and LPS induce higher level TGF-*β*1 in CFA-immunized Sprague Dawley rats splenocytes in vitro and LPS added to normal synoviocytes causes higher amount of TGF-*β*1. Increased levels of IL10 in serum were found in patients with RA [35]. In addition to losing anti-inflammatory functions, IL-10 can acquire proinflammatory functions. Evidence exists that interleukin- (IL-) 10 family cytokines may be involved in the pathogenesis of RA [36]. Increased serum levels of many cytokines were indeed found in other rheumatic diseases: notably psoriatic arthritis (IL-6, IL-7, IL-10, and IFN-*γ*, TGF-*β*, or TNF-*α*) suggesting that such rises may reflect inflammation rather than being disease specific [37–40].

Taken together, CFA-immunized Sprague Dawley rats are characteristic of a rapid onset of inflammation and higher values for common RA parameters, as shown by splenocyte cytokine analysis, the most accurate prognostic marker of human RA. This model is very useful for assessing the efficacy of various arthritis treatment protocols. Therefore, PFS and Periplocin can prophylactically treat autoimmune arthritis not only by controlling the systemic autoimmune responses but also by controlling local inflammation and bone destruction of the joints.

## Figures and Tables

**Table 1:** Primers for target genes.

Gene name	Primers	bp	Cycle	(°C)
IL-6	F: GCAAGAGACTTCCAGCCAGTT	229	35	54
	R: CATCATCGCTGTTCATACAATCA			
TGF-*β*1	F: CTCAACACCTGCACAGCTCC	349	35	56
	R: ACGATCATGTTGGACAACTGCT			
IFN-*γ*	F: GGAACTGGCAAAAGGACGGT	209	35	55
	R: GGGTTGTTCACCTCGAACT			
T-bet	F: TCCTGTCTCCAGCCGTTTCT	122	35	55
	R: CGCTCACTGCTCGGAACTC			
GATA3	F: CCCCATTACCACCTATCCGC	356	35	55
	R: CTCCGTTAGCGTTCCTCCTC			
C-Jun	F: ATGACTGCAAAGATGGAAACG	376	35	50.4
	R; TATTCTGGCTATGCAGTTCAG			
IL-22	F: GTTCCGAGGAGTCAAAGCCAA	117	35	60
	R: ACCTCCTGCATGTAAGGCTG			
IL-13	F: CACAAGACCAGAAGACTTCCCT	172	35	58
	R: ACATCCGAGGCCTTTTGGTT			
*β*-Actin	F: GGAGATTACTGCCCTGGCTCCTA	150	35	58.2
	R: GACTCATCGTACTCCTGCTTGCTG			
